# Supplementary material for: Measuring instability in chronic human intracortical neural recordings towards stable, long-term brain-computer interfaces
Source: Commun Biol. 2024 Oct 21;7:1363. doi: 10.1038/s42003-024-06784-4 (PMC11494208; doi:10.1038/s42003-024-06784-4)
Supplement: Supplementary file 2 — Supplementary Material [file 42003_2024_6784_MOESM2_ESM.pdf]

Supplementary materials for Measuring instability in chronic human intracortical neural recordings towards stable, long-term brain-computer interfaces

Tsam Kiu Pun, Mona Khoshnevis, Thomas Hosman, Guy H. Wilson, Anastasia Kapitonava, Foram Kamdar, Jaimie M. Henderson, John D. Simeral, Carlos E. Vargas-Irwin, Matthew T. Harrison**, Leigh R. Hochberg**

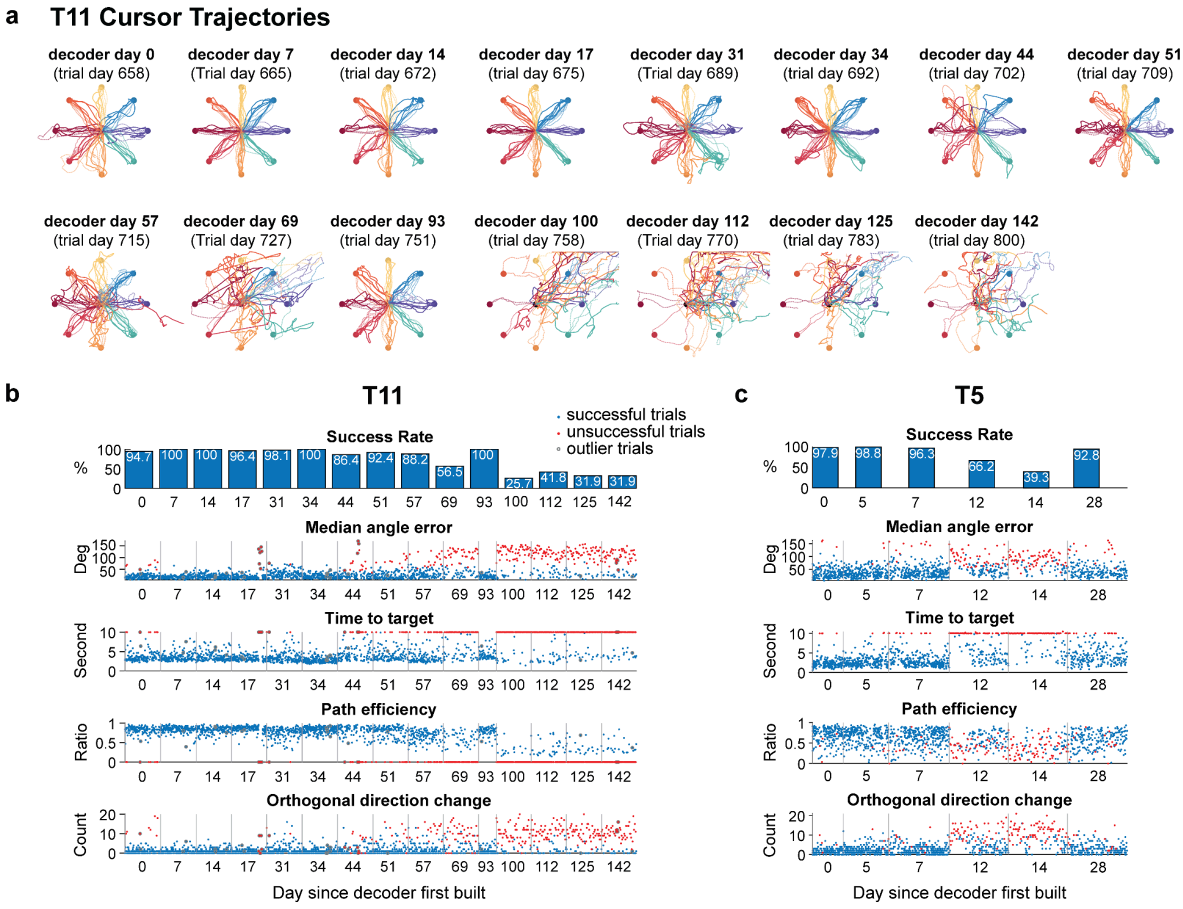

Supplementary Figure 1. Cursor task performance using a fixed decoder

(a) Adapted from [51]. Cursor trajectories in the first 5-min center-out task for selected trial days. Colors correspond to different target locations. A solid line indicates the cursor trajectory of a trial going from center to a peripheral target, and a dotted line indicates cursor trajectory of a trial from a peripheral target back to the center. Towards later sessions where the decoder began to degrade for T11, there was a consistent directional bias to the upper right corner. Trials that required the cursor to move to the bottom-left targets tended to fail.

(b) Adapted from [51]. Overall trial success rate per trial day, and other trial-to-trial performance metrics across trial days for T11. Each dot represents either a successful trial in blue or an unsuccessful trial in red where the cursor fails to reach the target before a 10-s timeout. Trials with gray circles are outlier trials with significant noise in the recordings. Cursor control is assessed using the following performance metrics: trial success rate, average trial success, angle error; time to target, time to reach to the target per trial; path efficiency, which is the ratio of the actual trajectory length to the Ideal straight-line path (best = 1) in a trial; and the number of orthogonal direction changes (ODC), where the cursor reversed away from or then back toward the target, which quantifies the path consistency towards the target (best = 0).

The decoder achieved an average of 93.8% success rate in reaching and clicking the cued target in the first 11 sessions in the three months (trial day 658-751). But it subsequently degraded to 33.1% in later sessions (trial day 758-800). On average per trial, early sessions (658-751) demonstrated higher performance than later sessions (758-800) in terms of median AE (early: 26.8° ± 22.6°; later: 88.4° ± 46.1°; $p < 0.001$; Wilcoxon rank sum), time to target (early: 4.02 ± 1.98 s; later: 8.01 ± 3.04 s; $p < 0.001$), path

efficiency (early: 0.79 ± 0.12; later: 0.38 ± 0.17; $p < 0.001$; excluding unsuccessful trials) and orthogonal directional change (early: 1.82 ± 2.82; later: 7.40 ± 5.35; $p < 0.001$).

(c) Same performance metrics across trial days as **(b)** for T5. There were no outlier trials that had significant noise events. Trial success rate in the first three sessions was 97.6% compared to 71.6% in later sessions. The first three sessions demonstrated higher average performance than the later three sessions in terms of AE (early: 39.6° ± 23.9°; later: 58.8° ± 31.7°; $p < 0.001$), time to target (early: 2.87 ± 1.71 s; later: 5.90 ± 3.12 s; $p < 0.001$), path efficiency (early: 0.66 ± 0.20; later: 0.52 ± 0.22; $p < 0.001$; excluding unsuccessful trials) and orthogonal directional change (early: 2.21 ± 2.08; later: 5.59 ± 4.65; $p < 0.001$).

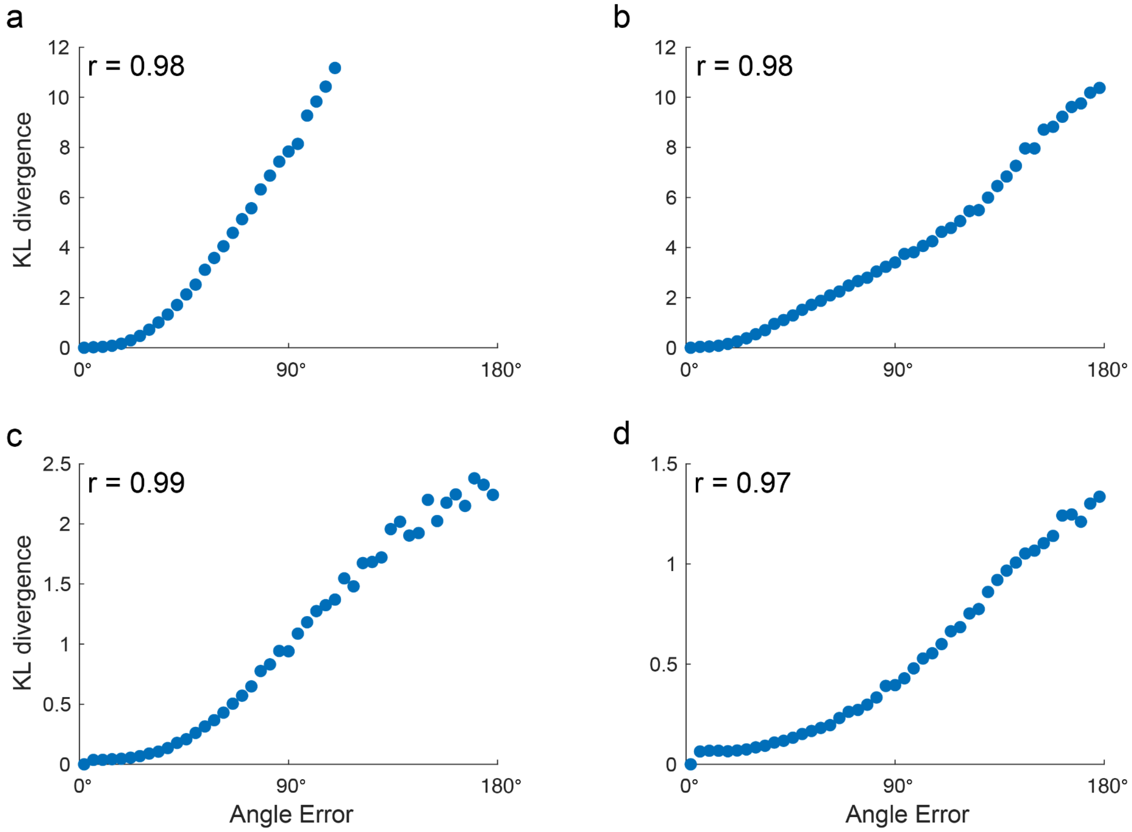

Supplementary Figure 2. A linear relationship between KLD and angle error in simulated noise or model drift. $KL(p(Y|0 < AE < 4), p(Y|\theta - 2 < AE < \theta + 2))$ changes with angle error, θ (see **Supplementary methods**). **(a-b)** displayed case I where noise was applied without additional model drifts, and **(c-d)** displayed case II where added model drifts (tuning changes) were applied. In **(a), (c)**, we use a low model noise ($\sigma^2 = 4$), while in **(b), (d)**, a higher model noise ($\sigma^2 = 25$) is applied. Pearson correlation coefficient was shown in each panel.

Both simulated noise and model drift result in a linear relationship between $KL(p(Y|0 < AE < 4), p(Y|\theta - 2 < AE < \theta + 2))$ with θ. Additionally, a higher level of noise corresponds to increased angle error. The difference in coverage of angle errors is notable: **(c)** spans the entire possible range (from 0° to 180°), while panel **(a)** only extends up to 110°. This discrepancy potentially underscores the contribution of model drifts to the KLD, surpassing mere noise effects.

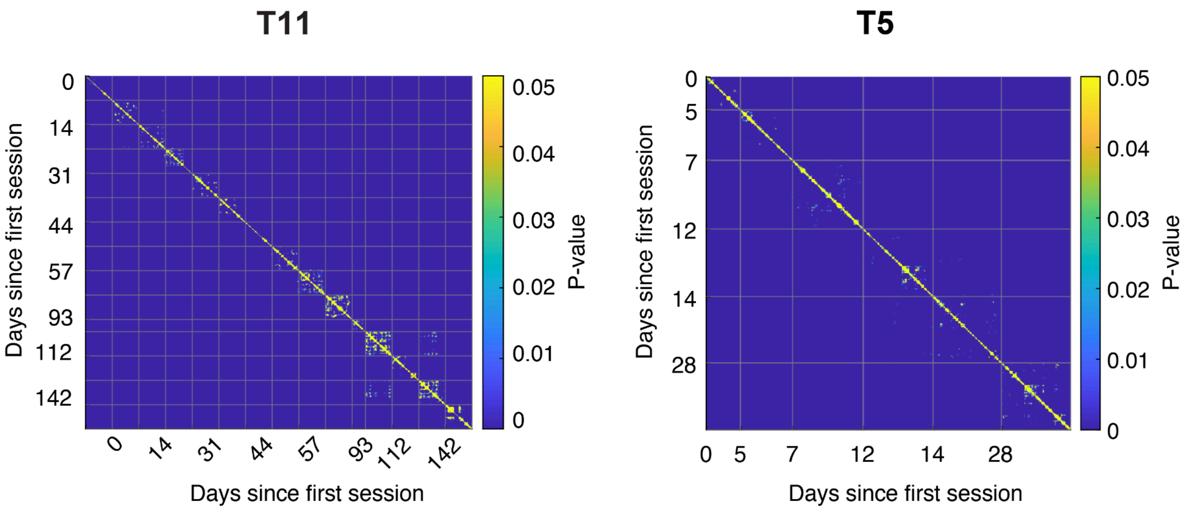

Supplementary Figure 3 Multiple hypothesis testing with Bonferroni corrected p-value. Bonferroni corrected p-value between pairs of kinematics-neural data from time bins from sessions was computed, for T11 and T5 (see **Supplementary methods**). The compared data are each 60 s sliding window updated every 10 s. The data used to obtain the PCA transform are the same as **Fig. 1c, 2,** and **5**, namely, time bins from the first session (T11) or first two sessions (T5) that have AE < 4°. Neural features are projected to the top M=5 PCs. The hypothesis test suggests the presence of model drifts on almost every pair, except the neighbor ones along the diagonal, for both participants.

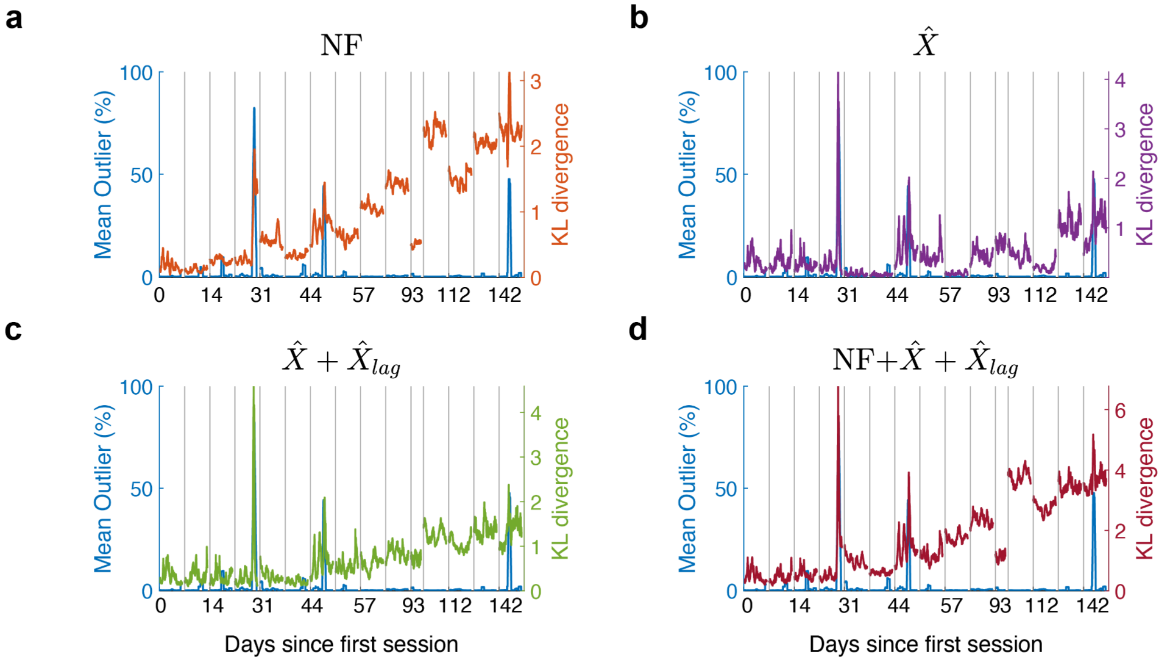

Supplementary Figure 4. Sharp spikes in the KLD correspond to recording outliers, Participant T11. Mean outliers over each sliding window of 60 s were shown on the left y-axis. Outliers at every 20 ms time bin were defined by the maximum percentage of noise events across electrodes during a session. Noise events can be attributed to drop packets and bit flips from data transmission and large recording signal instances (>8 standard deviations from mean). Gray lines indicate the beginning of the session. **(a)** Across all sessions, large spikes were observed in the KLD on low-dimensional neural data (same as **Fig. 1a**) at the occurrence of three large abrupt momentary surges of outlier events (around end of day 17, middle of day 44 and 142). Large spikes in KLD using **(b)** decoded velocity, $\hat{x}$, or **(c)** $\hat{x}$ and its lag, $\hat{x}_{lag}$. were presented for the first two outlier surges on day 17 and 44. **(d)** When KLD was computed on low-dimensional neural data along with decoded velocity and its lag, the large spikes on day 17 and 44 was prominent whereas the spike on day 142 is less prominent than **(a)**. This suggests that large noisy events were reflected in the recorded neural data, and can affect the decoded cursor movement but only momentarily. And KLD appeared to be sensitive to these large sudden deviations due to noise.

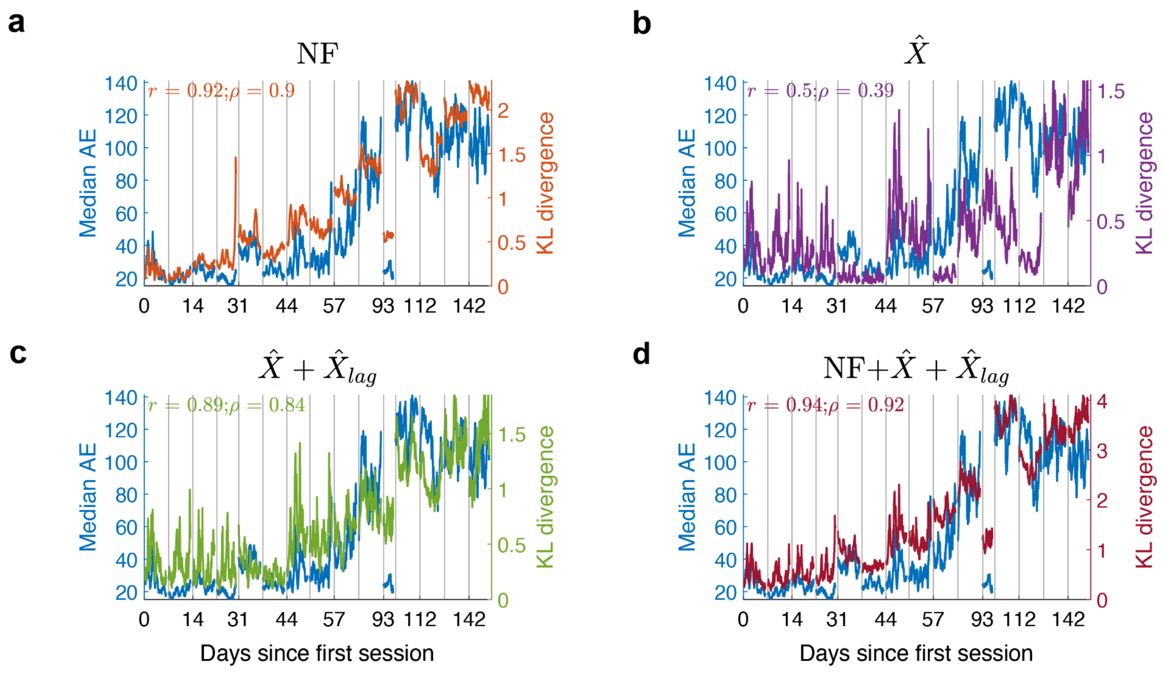

Supplementary Figure 5. KLD excluding the outlier trials, Participant T11. When excluding trials with more than 5% mean outliers across the trial duration, KLD was also strongly correlated with the online median AE, and even higher than when outlier trials were included. This reflects that MINDFUL was still capable of monitoring long-term model drift that leads to degrading performance, in addition to detecting outlier events.

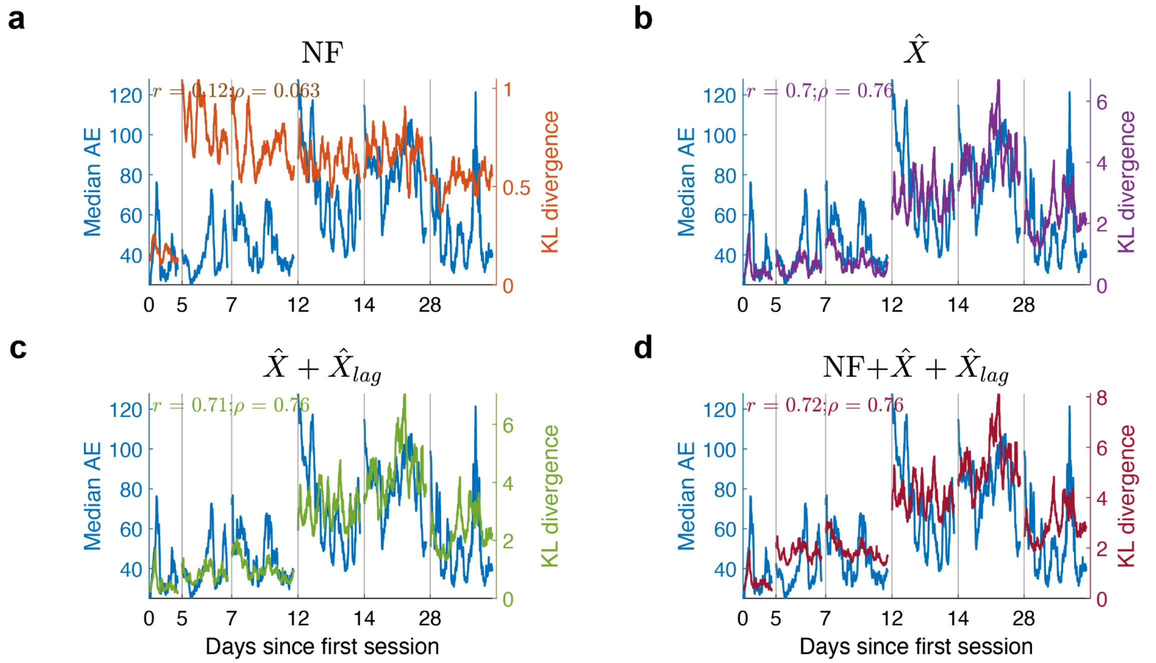

Supplementary Figure 6. KLD using the first session as the reference, Participant T5. (a) KLD (right y-axis) overlaid onto median angle error (left y-axis in blue) across all recorded sessions T5. Distributions were estimated from low-dimensional neural data after PCA. Neural features on the first session were selected for the reference distribution. Only time steps when AE < 30° were sub-selected as the reference. Subsequent neural distributions and median AE were updated every 1 s over a 60 s sliding window. Pearson r, and Spearman rank correlation coefficients ρ, between KLD and median AE were calculated. **(b)** KLD between distributions of just decoded directional vectors, $\hat{x}$, not neural data. **(c)** KLD between distributions of just $\hat{x}$ and $\hat{x}_{lag}$. **(d)** KLD between distributions of a combination of low-dimensional neural data, decoded directional vectors, $\hat{x}$, and $\hat{x}_{lag}$.

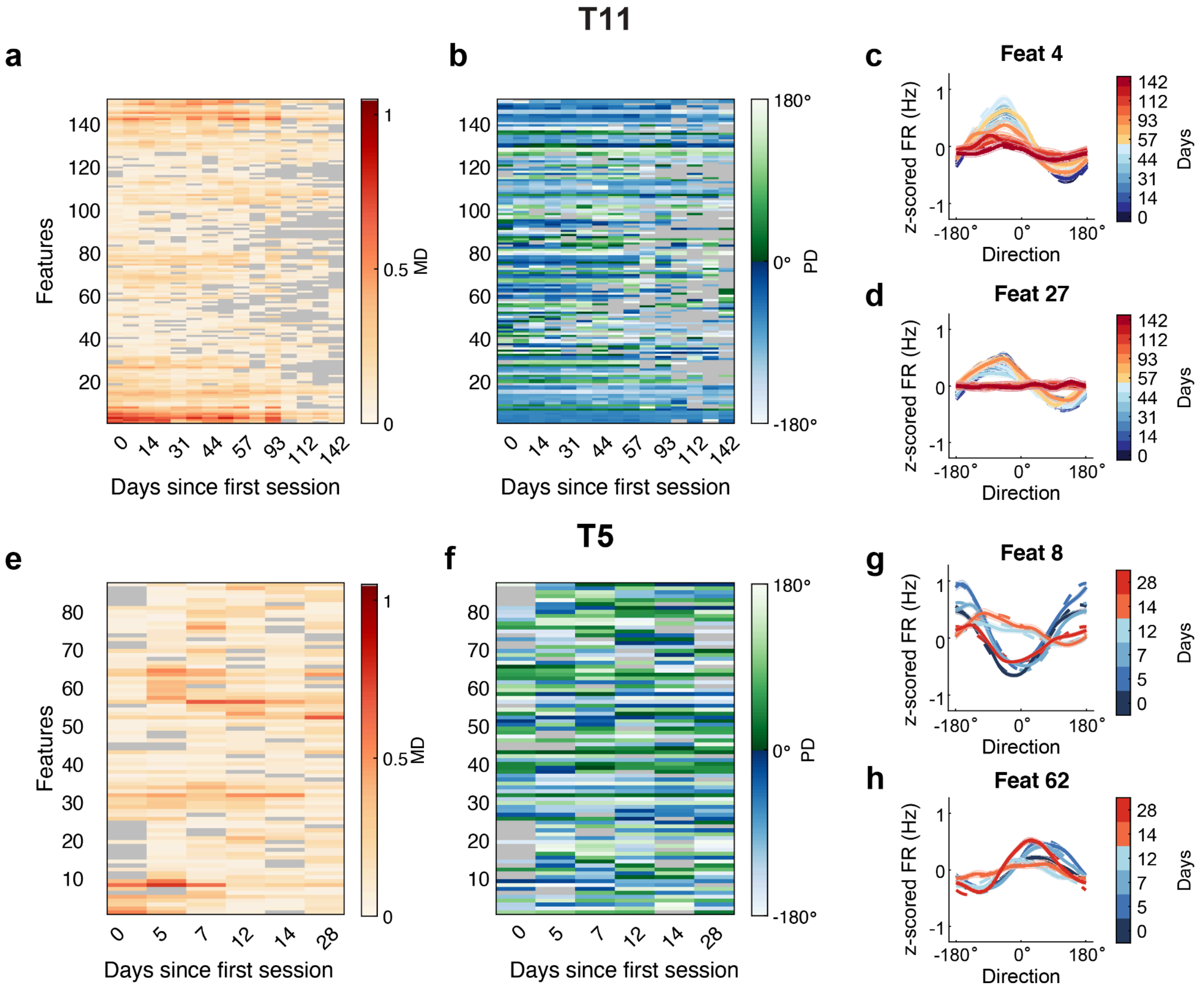

Supplementary Figure 7. Modulation depth (MD) and preferred direction (PD) of individual features

(a) MD and **(b)** PD of significantly tuned features of T11. Features are sorted using the same hierarchical ordering as **Fig. 3(a)** and **3(b)**. **(c-d)** Example neural model drift of two features across sessions of T11 along with empirical firing rate. Dotted lines correspond to cosine tuning models fit within each session as shown in Figure 4d and 4e. Solid lines are empirical firing rates estimated using Nadaraya-Watson kernel regression[1], along with the 95% confidence intervals in shaded bands. Line colors denote the session day from light to dark progression. **(e)** MD and **(f)** PD of significantly tuned features of T5. **(g-h)** Example neural model drift of two features across sessions of T5 as shown in **Fig. 3(i) and 3(j)**.

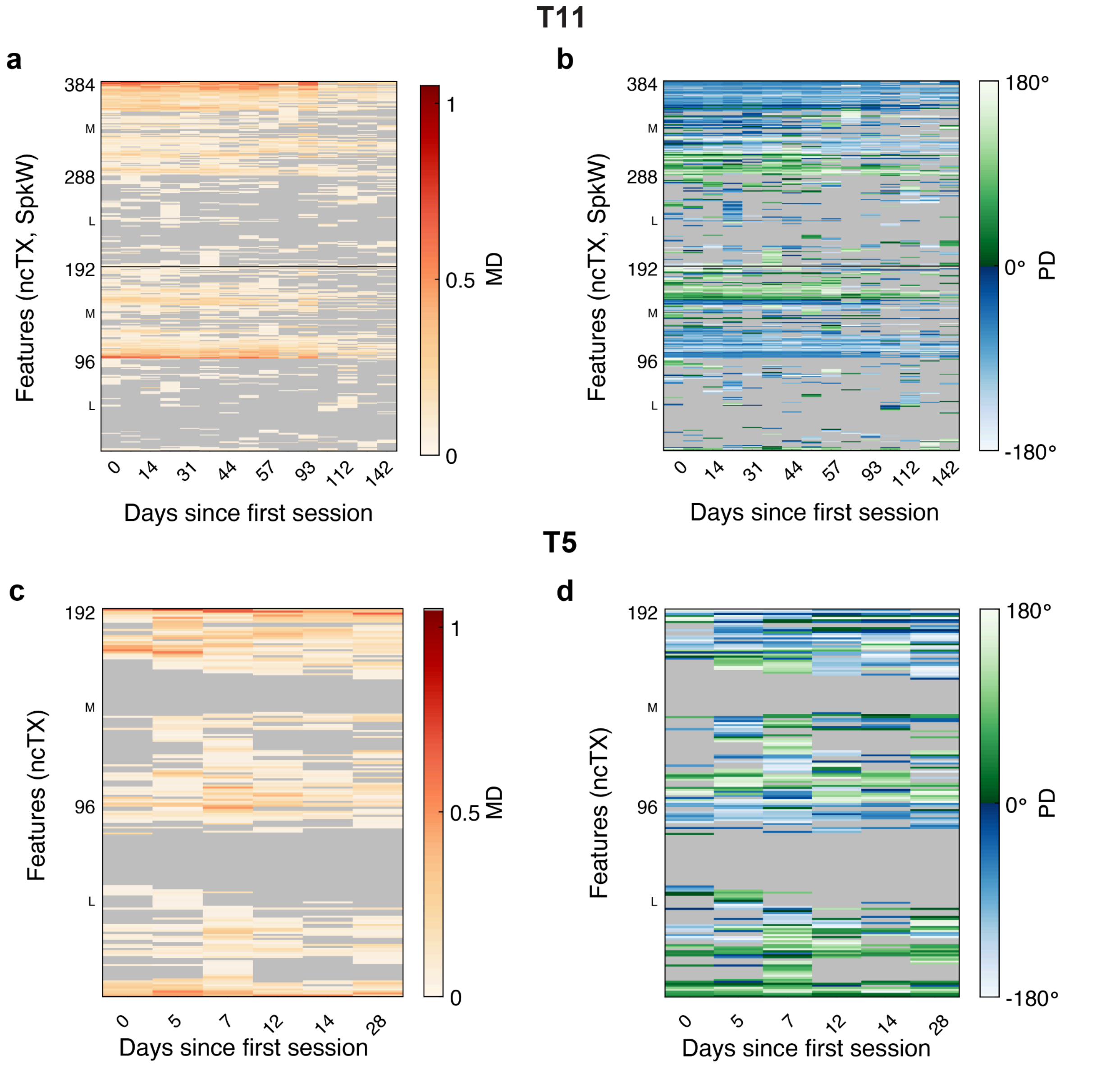

Supplementary Figure 8. Modulation depth (MD) and preferred direction (PD) of all feature tunings
Tunings for all features used for online decoding were estimated. **(a)** MD and **(b)** PD of all features used in online decoding across all sessions for T11. Both tuning maps were ordered using the same hierarchical clustering methods. Hierarchical clustering was applied per array (L: lateral, M: medial) per feature type (ncTX: threshold crossings, SpkW: spike power). Gray out features were not significantly tuned ($p > 0.05$). **(c)** MD and **(d)** PD of all features sorted by hierarchical clustering per array for T5.

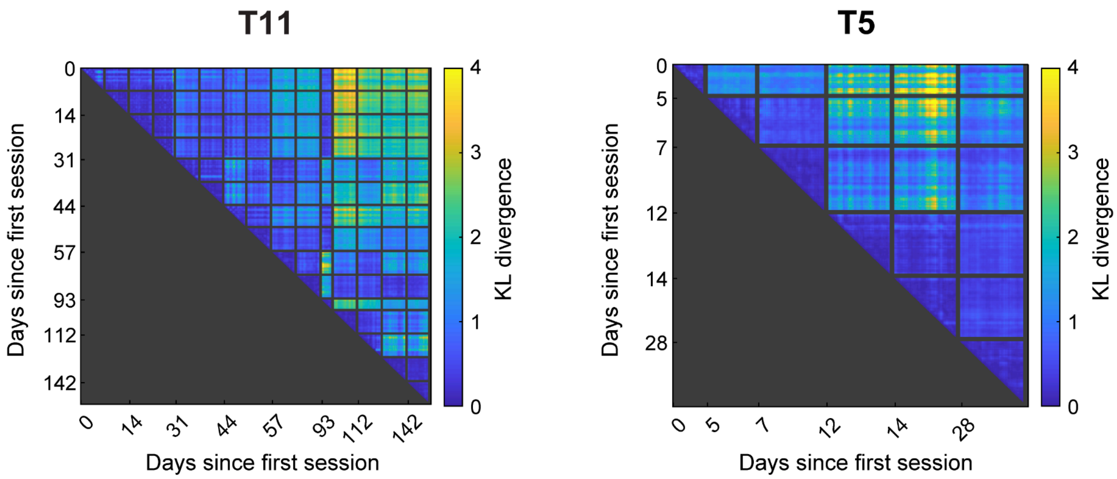

Supplementary Figure 9. Pairwise KLD between sessions

For each day, distributions were estimated from low-dimensional neural features, in combination with decoded output, $\hat{X}$ and $\hat{X}_{lag}$, from overlapping time segments using a 60 s sliding window updated every 10 s. Outlier trials were excluded. Only the upper triangle matrix is shown as KLD is computed between distributions estimated from two time segments where the first segment precedes the second segment chronologically.

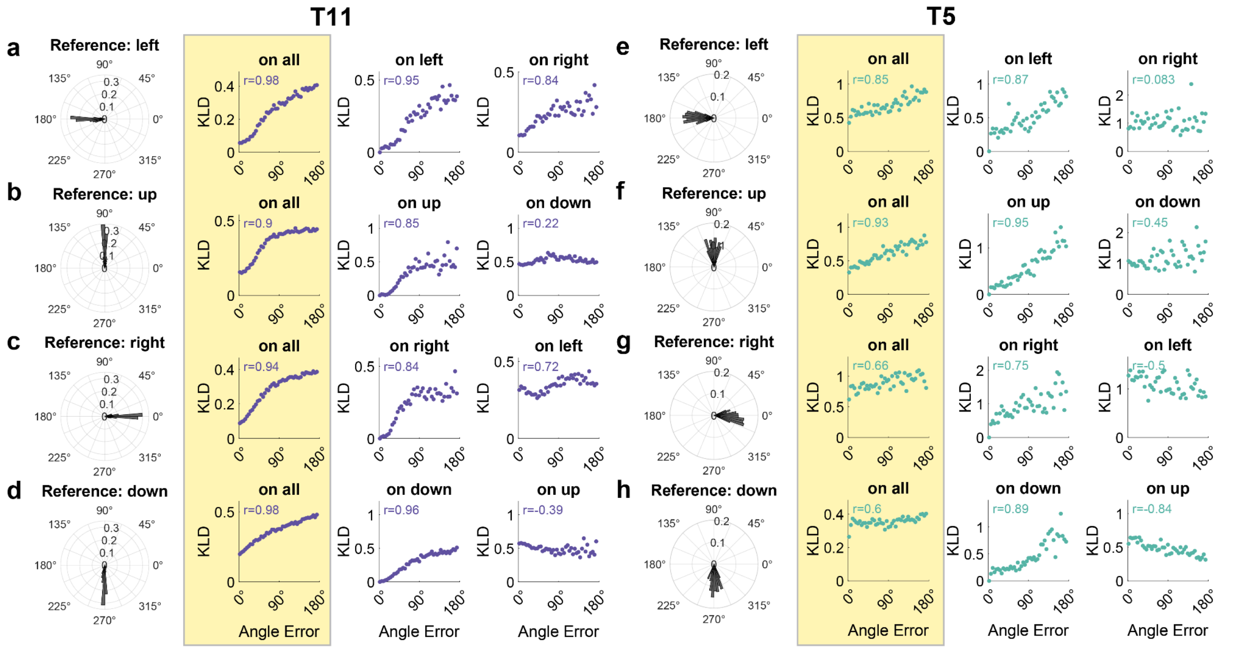

Supplemental Figure 10. MINDFUL is robust to reference data: KLD calculated using reference sub-selecting based on direction correlates with AE when compared to distributions not sub-selecting or sub-selecting based on the same direction, but is less correlated to distributions sub-selecting based on the opposite directions.

As in **Figure 1b**, samples of neural features were grouped according to decoder performance (AE), combining all sessions. The reference was neural features at time instances with AE < 4° and conditioned to decoded movement going within ±22.5° towards one of the four directions centered at **(a)** 0° (left), **(b)** 90° (up), **(c)** 180° (right) and **(d)** 270° (down) for T11, similarly **(e-h)** for T5. The left polar histogram on each panel indicates the distribution of such sub-selecting references. In each panel, the same conditional references were compared to the other 44 bins with increasing AE intervals, under three conditions: (i) “on all” refers to distributions that include all time bins of AE within each given AE interval. “on up/down/left/right” refers to subsampled time steps within the AE intervals that are also moving towards the specified direction (either (ii) same as reference direction or (iii) opposite direction).

The polar histogram appears to be narrower to the center direction for T11 because of the task design: T11 was performing a center-out-and-back task versus T5 was performing a random target task. Under condition (ii), because the reference and the first target distribution are the same (both AE < 4° going in the same direction), KLD starts at 0. Under the other two conditions, since the first target distribution is different, KLD starts at a non-zero value. When compared to target distribution (i) no sub-selecting or (ii) sub-selecting to the same direction as the reference, KLD still linearly tracks with performance, but for (ii), the range of KLD appears to be bigger for T5, and the pearson correlation is higher than (i) for T5 but not for T11. As for (iii) where the compared distributions are going in the opposite direction, there is relatively much less correlation for both T11 and T5, except in panel **(a)**.

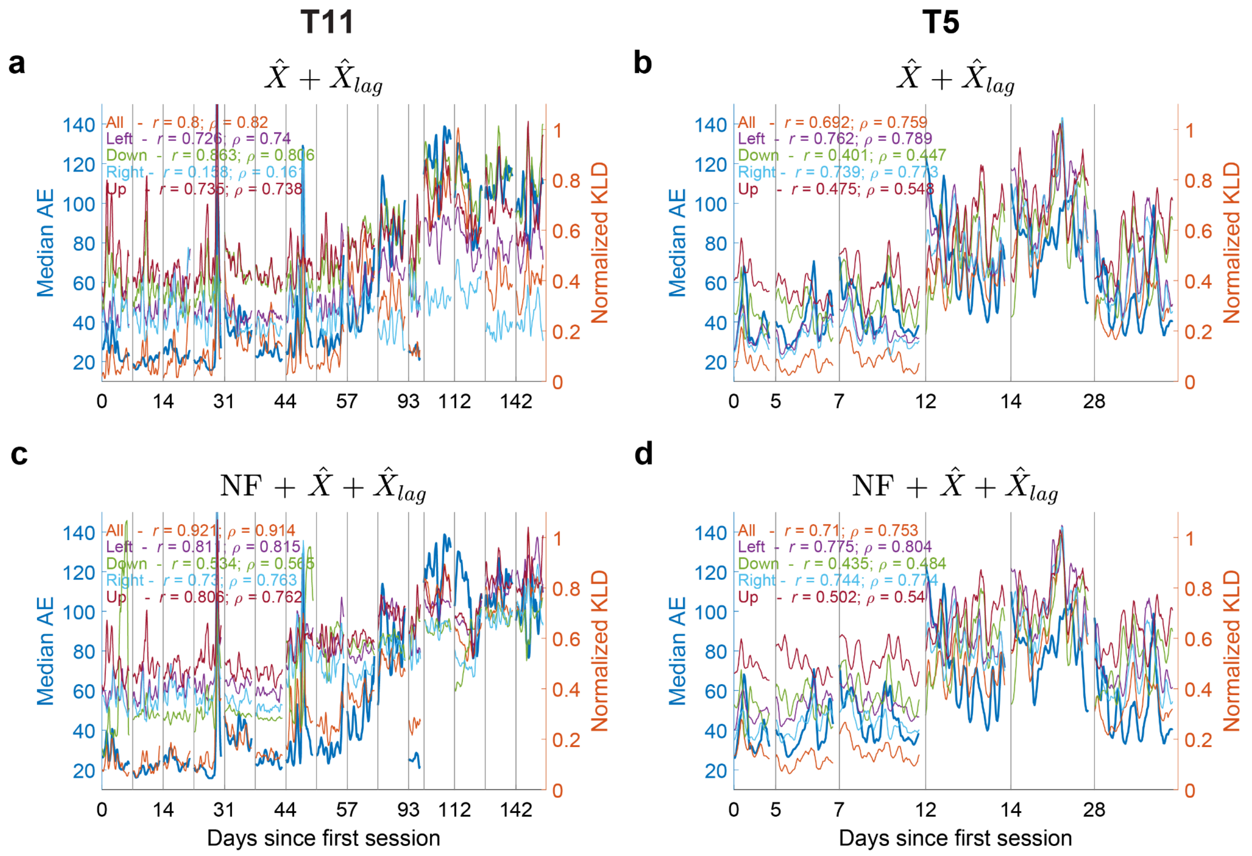

e

	$\hat{X} + \hat{X}_{lag}$					$\mathrm{NF} + \hat{X} + \hat{X}_{lag}$				
	**All**	**Left**	**Down**	**Right**	**Up**	**All**	**Left**	**Down**	**Right**	**Up**
**T11**	1.25	2.5	3.07	2.27	3.1	4.58	8.08	26.44	10.04	8.87
**T5**	5.45	16.15	8.36	23.17	4.93	6.28	20.24	10.29	26.97	7.18

Supplemental Figure 11. Uneven sampling of movement directions in the reference distribution decreases correlation between KLD and AE

(a) The KLD between distributions of X and Xlag overlaid onto median AE, for T11 and **(b)** for T5. “All” in orange indicates when the reference is not sub-selecting based on direction (same as **Fig. 2b**), and other colors indicate the directions where the reference is sub-selecting on. **(c)** The KLD between distributions of the combination of derived neural features, decoded directional velocity and its lagged velocity, overlaid onto median AE, for T11 and **(d)** for T5. “All” also indicates using a reference that was not sub-selecting based on direction (as in **Fig. 1c**) Colors of the plots are consistent with the upper panels.

For easier comparison, for each line, all KLDs are normalized by scaling down by the 99.5th percentile of all its data points and the scaling values are shown in the table on panel **(e)**.

The different choices of reference, sub-selected by direction, still highly correlates with AE, although not as highly as the “all” reference for T11, and “Left” and “Right” correlate slightly more highly than the “all” reference for T5. However, the scaling factor is much higher for some directions, and the KLD baseline is also higher.

	Fig. 2		Higher dim. in NF		No reference sub-selection		No z-score in NF		No PCA in NF	
Reference sub-selection	AE < 4°		AE < 4°		all timesteps		all timesteps		all timesteps	
PCA: top PCs	5		10		5		5		-	
**T11**										
Correlations	r	ρ	r	ρ	r	ρ	r	ρ	r	ρ
NF + $\hat{X}$ + $\hat{X}_{lag}$	0.926	0.913	0.923	0.898	0.923	0.838	0.319	0.602	0.093	0.522
NF	0.91	0.9	0.905	0.877	0.887	0.774	0.325	0.596	0.09	0.52
NF + $\hat{X}$	0.878	0.882	0.9	0.882	0.902	0.801	0.321	0.596	0.091	0.52
$\hat{X}$	0.464	0.407	0.464	0.407	0.69	0.748	0.69	0.748	0.69	0.748
$\hat{X}$ + $\hat{X}_{lag}$	0.819	0.84	0.819	0.84	0.827	0.844	0.827	0.844	0.827	0.844
**T5**										
correlations	r	ρ	r	ρ	r	ρ	r	ρ	r	ρ
NF + $\hat{X}$ + $\hat{X}_{lag}$	0.719	0.759	0.72	0.758	0.706	0.722	0.387	0.375	0.025	0.152
NF	0.587	0.596	0.53	0.532	0.474	0.498	0.244	0.289	0.016	0.129
NF + $\hat{X}$	0.715	0.768	0.716	0.765	0.717	0.775	0.391	0.377	0.019	0.188
$\hat{X}$	0.704	0.763	0.704	0.763	0.708	0.78	0.708	0.78	0.708	0.78
$\hat{X}$ + $\hat{X}_{lag}$	0.702	0.765	0.702	0.765	0.702	0.749	0.702	0.749	0.702	0.749

Supplementary Table 1. Ablation analysis for MINDFUL
Higher dimension in neural features (NF): Using 10 PCs rather than 5 when estimating the mean and variance of the high-dimensional neural data did not make a difference in the correlation between the KLD and AE.

No reference sub-selection: Without sub-selection of time steps with low AE (<4°) in the reference distribution, correlations decreased slightly for cases where NF were used as part of the input features to calculate the KLD. However, correlations were higher for T11, and similar for T5 when only using $\hat{X}$ or $\hat{X}$ + $\hat{X}_{lag}$ as the features.

No z-score in NF: Without z-scoring neural data, mean shifts were more pronounced in measuring distribution difference using KLD (not shown). This resulted in low correlation to online performance since mean shifts were accounted for with adaptive z-scoring and bias removal for T11 and T5 respectively.

No PCA in NF: When not applying PCA, there was little-to-no correlation in terms of Pearson's coefficient for both T11 and T5 in cases where NF were used. This could be due to the fact that directly estimating the distribution of high dimensional data becomes difficult as the number of samples in a 60s sliding window is relatively limited when compared to the number of dimensions. It also substantially increases the computational power required.

Supplemental methods

1 Linear KLD-AE relationship using simulated models

The timescale of how a change persists matters in clinical settings. Because if the dominant cause of the linear relationship demonstrated in Fig 1b is intermittent noise, which occurs at sub-second timescale and does not contain any persistent changes, one cannot predict performance by combining time bins of neural activity in any way without knowing the performance in the first place. However, if the dominant cause comes from changes that persist over time, i.e. model drift, any contiguous group of time bins of neural data may define a collection where its statistical properties will reflect a shared source of error.

To study how each noise and model drifts contributes to KLD, consider the following linear regression encoding model for neural data given kinematics,

$$Y = AX + Z. \tag{1}$$

In this equation, Y represents the matrix of neural features (size $d \times n$), X is the movement intentions (size $l \times n$), and A denotes the regression coefficients (tuning) matrix (size $d \times l$). Here, we used $d = 50$, and $l = 2$. We assume a multidimensional Gaussian noise Z in this model, which is independent of X and follows a distribution of $N_d(\mathbf{0}, \sigma^2 I)$.

For this linear model, under the assumption that $X \sim N(\mathbf{0}, I)$, the optimal decoder $\mathbb{E}[X|Y]$ is $\hat{X} = BY$, where $B = A^T(AA^T + \sigma^2 I)^{-1}$. We denote $AE = \text{angle}(X, \hat{X})$ as the angle error of this estimation.

We generate synthetic data by considering two scenarios:

Case (I): No added model drifts:

We simulate neural features using the encoding model in (1) for $n = 10^4$ timesteps for 50 simulated sessions. Throughout these sessions, the parameters of the model A and σ^2 remain constant. This process yields the matrix $\overline{Y}$, representing neural features of size $d \times 50n$. After decoding $\overline{Y}$ using the optimal decoder $B = A^T(AA^T + \sigma^2 I)^{-1}$, we calculate the angle error for each time step across sessions.

Next, we estimated two conditional distributions:

1) The target distribution of neural features $\overline{Y}$ conditioned on a specific angle error range $\theta - 2° < \overline{AE} < \theta + 2°$, and,

2) The reference conditional distribution of neural features $\overline{Y}$ given good performance, where $0° < \overline{AE} < 4°$.

We compute the KLD for varying angle errors $\theta \in \{2°, 6°, 10°, \cdots, 178°\}$.

Case (II): Model drifts with tuning changes is added:

The process remains akin to the one in case (I), except for a daily alteration in the parameter A of the encoding model. This parameter changes randomly each session day from the original tuning matrix A (used to build the decoder $B = A^T(AA^T + \sigma^2 I)^{-1}$) to a newly chosen tuning matrix A^*. This alteration is achieved using a linear combination of the original matrix and the

new tuning matrix: $\lambda A + (1-\lambda)A^*$, where λ is uniformly chosen at random from $[0,1]$. Despite this daily change in the encoding model, the neural features $\overline{Y}$ across all days is still decoded using the same decoder B, which was specifically optimized for $\lambda = 0$. (The other parameter of the encoding model (σ^2) remains unchanged daily). Similar to case (I), the KLD between $p(\overline{Y}|0^\circ < \overline{AE} < 4^\circ)$ and $p(\overline{Y}|\theta - 2^\circ < \overline{AE} < \theta + 2^\circ)$ (both assumed Gaussian) for varying θ is calculated.

This procedure captures how the encoding model's parameter changes affect the distribution of neural features, even when decoded using an optimal decoder designed for a stable parameter ($\lambda = 0$). The KLD is computed across different angle error ranges (θ) to assess the impact of these parameter variations. In contrast, in case (I), where there is no change in the encoding model parameters, the KLD is solely affected by the instantaneous noise present in the system.This distinction allows us to discern how variations in the encoding model parameters specifically contribute to the differences observed in the neural data distribution, separate from the impact of mere noise fluctuations.

2 Hypothesis test for model drifts

As another method to detect model drifts in our probabilistic encoding model, we use a multiple hypothesis test described as below.

For two kinematics-neural pairs (X, Y) and $(\overline{X}, \overline{Y})$, where Y and $\overline{Y}$ are d-dimensional neural features of sizes $d \times T$ and $d \times \overline{T}$ respectively, and X and $\overline{X}$ are movement intention matrices of size $2 \times T$ and $2 \times \overline{T}$, we assume the linear regression models below:

$$Y = A\,X + Z$$

$$\overline{Y} = \overline{A}\,\overline{X} + \overline{Z}$$

where A and $\overline{A}$ are the tuning matrices, and each column of Z ($\overline{Z}$) is independently distributed as $N_d(\mathbf{0}, \Sigma)$ ($N_d(\mathbf{0}, \overline{\Sigma})$).

The null hypothesis which we want to test is $H_0 : (A = \overline{A}, \Sigma = \overline{\Sigma})$. Under the assumption that Z and $\overline{Z}$ are independent, we define two hypothesis tests for each dimension of neural features separately[2]. In other words, for each dimension $1 \leq j \leq d$, since T ($\overline{T}$) observations are assumed to be independent, the models above can be written as

$$Y_j = A_j X + z$$

$$\overline{Y}_j = \overline{A}_j \overline{X} + \overline{z}$$

where $Y_j(\overline{Y}_j)$ and $A_j(\overline{A}_j)$ denote the j's row of $Y(\overline{Y})$ and $A(\overline{A})$, and $z(\overline{z})$ is a $1 \times T$ $(1 \times \overline{T})$ matrix of noise distributed as $N_T(\mathbf{0}, \Sigma_{jj} I_T)(N_{\overline{T}}(\mathbf{0}, \overline{\Sigma}_{jj} I_{\overline{T}}))$. ($\Sigma_{jj}$ is the (j,j) entry of Σ.) Now for each $1 \leq j \leq d$, we use test statistics[2] to find p_j^A and p_j^Σ, p-values associated to the individual tests $H_{0_j}^A : A_j = \overline{A}_j$, and $H_{0_j}^\Sigma : \Sigma_{jj} = \overline{\Sigma}_{jj}$, respectively in the following way:

$$p_j^A(X, Y_j, \overline{X}, \overline{Y}_j) = 1 - I_{\frac{2a}{2a + (T + \overline{T} - 4)}}(1, \frac{T + \overline{T} - 4}{2})$$

for

$$a = \frac{T + \overline{T} - 4}{2}\left(\frac{||(Y_j X' + \overline{Y}_j \overline{X}')(XX' + \overline{X}\,\overline{X}')^{-1}||^2}{||Y_j - Y_j X'(XX')^{-1}X||^2 + ||\overline{Y}_j - \overline{Y}_j \overline{X}'(\overline{X}\overline{X}')^{-1}\overline{X}||^2} - 1\right)$$

where $||.||$ is the Euclidean norm of a vector and I is the regularized incomplete beta function.

Also,

$$p_j^{\Sigma}(X,Y_j,\overline{X},\overline{Y}_j) = 2\min\Big(I_{\frac{(\overline{T}-2)b}{(\overline{T}-2)b+(T-2)}}(\frac{\overline{T}-2}{2},\frac{T-2}{2}), 1 - I_{\frac{(\overline{T}-2)b}{(\overline{T}-2)b+(T-2)}}(\frac{\overline{T}-2}{2},\frac{T-2}{2})\Big)$$

where

$$b = \frac{T-2}{\overline{T}-2}\,\frac{||\overline{Y}_j - \overline{Y}_j\overline{X}'(\overline{X}\overline{X}')^{-1}\overline{X}||^2}{||Y_j - Y_jX'(XX')^{-1}X||^2}$$

Then the the p-value defined to test $H_0 : (A = \overline{A}, \Sigma = \overline{\Sigma})$ is:

$$p^*(X,Y,\overline{X},\overline{Y}) = \min\Big\{1, 2d\min\Big\{\min_{j=1}^{d} p_j^A(X,Y_j,\overline{X},\overline{Y}_j), \min_{j=1}^{d} p_j^{\Sigma}(X,Y_j,\overline{X},\overline{Y}_j)\Big\}\Big\}$$

This Bonferroni corrected P-value can be easily shown to be valid for H_0 in the sense that $\mathbb{P}(p^*(X,Y,\overline{X},\overline{Y}) \leq \alpha) \leq \alpha$ for all $\alpha \in [0,1]$ under H_0.

Remark: When Σ and $\overline{\Sigma}$ are not diagonal, the Bonferroni correction described above does not have the power to detect whether covariances between error terms among different dimensions are equal or not. In other words, If we have $A = \overline{A}$ and $\Sigma_{jj} = \overline{\Sigma}_{jj}$ for all $1 \leq j \leq d$, but the there exist $1 \leq j \neq j' \leq d$ such that $\Sigma_{jj'} \neq \overline{\Sigma}_{jj'}$, then the offered Bonferroni correction would not reject that.

Remark: There are possible scenarios in which the distribution of neural features by itself is not informative enough about the change in the encoding model, unless we have already fixed the movement intention. This is why inspection of model drifts on the conditional distribution $\mathbb{P}(Y|X)$ using the procedure above can work better on those cases.

References

[1] Härdle, W. *Applied Nonparametric Regression*. *Cambridge University Press.* (1990).

[2] Toyoda, T. & Ohtani, K. Testing equality between sets of coefficients after a preliminary test for equality of disturbance variances in two linear regressions. *Journal of Econometrics*, vol. 31, 67–80 (1986).
