# Supplementary material for: Measuring instability in chronic human intracortical neural recordings towards stable, long-term brain-computer interfaces
Source: Commun Biol. 2024 Oct 21;7:1363. doi: 10.1038/s42003-024-06784-4 (PMC11494208; doi:10.1038/s42003-024-06784-4)
Supplement: Supplementary file 3 — reporting summary [file 42003_2024_6784_MOESM3_ESM.pdf]

nature portfolio

Corresponding author(s): Tsam Kiu Pun

Last updated by author(s): Aug 12, 2024

Reporting Summary

Nature Portfolio wishes to improve the reproducibility of the work that we publish. This form provides structure for consistency and transparency in reporting. For further information on Nature Portfolio policies, see our Editorial Policies and the Editorial Policy Checklist.

Statistics

For all statistical analyses, confirm that the following items are present in the figure legend, table legend, main text, or Methods section.

n/a	Confirmed	
☐	☒	The exact sample size (*n*) for each experimental group/condition, given as a discrete number and unit of measurement
☒	☐	A statement on whether measurements were taken from distinct samples or whether the same sample was measured repeatedly
☐	☒	The statistical test(s) used AND whether they are one- or two-sided<br>*Only common tests should be described solely by name; describe more complex techniques in the Methods section.*
☒	☐	A description of all covariates tested
☐	☒	A description of any assumptions or corrections, such as tests of normality and adjustment for multiple comparisons
☐	☒	A full description of the statistical parameters including central tendency (e.g. means) or other basic estimates (e.g. regression coefficient) AND variation (e.g. standard deviation) or associated estimates of uncertainty (e.g. confidence intervals)
☐	☒	For null hypothesis testing, the test statistic (e.g. *F*, *t*, *r*) with confidence intervals, effect sizes, degrees of freedom and *P* value noted<br>*Give P values as exact values whenever suitable.*
☒	☐	For Bayesian analysis, information on the choice of priors and Markov chain Monte Carlo settings
☒	☐	For hierarchical and complex designs, identification of the appropriate level for tests and full reporting of outcomes
☐	☒	Estimates of effect sizes (e.g. Cohen's *d*, Pearson's *r*), indicating how they were calculated

Our web collection on statistics for biologists contains articles on many of the points above.

Software and code

Policy information about availability of computer code

Data collection	The software for running the experimental tasks, data collection, and implementation of the real-time decoding system was custom developed using MATLAB and Simulink Real-Time (MathWorks, Natick, MA)
Data analysis	Data was analyzed using custom MATLAB code. Code is publicly available on Github (https://github.com/ewinapun/MINDFUL).

For manuscripts utilizing custom algorithms or software that are central to the research but not yet described in published literature, software must be made available to editors and reviewers. We strongly encourage code deposition in a community repository (e.g. GitHub). See the Nature Portfolio guidelines for submitting code & software for further information.

Data

Policy information about availability of data

All manuscripts must include a data availability statement. This statement should provide the following information, where applicable:

- Accession codes, unique identifiers, or web links for publicly available datasets
- A description of any restrictions on data availability
- For clinical datasets or third party data, please ensure that the statement adheres to our policy

All data required to reproduce the findings in this study are publicly available on Dryad (https://doi.org/10.5061/dryad.n2z34tn5s). The dataset contains intracortical neural signals recorded from both participants along with detailed information about the BCI behavior tasks and performance metrics

Research involving human participants, their data, or biological material

Policy information about studies with human participants or human data. See also policy information about sex, gender (identity/presentation), and sexual orientation and race, ethnicity and racism.

Reporting on sex and gender	This study included data from two participants, T5 and T11, who are both biological male and identifies as a man. This information was self-reported. No sex or gender based analyses were performed given there were only two participants, and were not the main focus of this study.
Reporting on race, ethnicity, or other socially relevant groupings	No variables relating to race, ethnicity or other socially relevant groupings were reported or analyzed.
Population characteristics	This study includes data from two participants who gave informed consent and was enrolled in the BrainGate2 Neural Interface System clinical trial (ClinicalTrials.gov Identifier: NCT00912041, registered June 3, 2009) - but note that this study does not report clinical trial results. Participant T5 is a right-handed man, 65 years old at the time of data collection, with a C4 AIS C spinal cord injury that occurred approximately 9 years prior to study enrollment. Participant T11 is a 37-year-old right-handed male with a C4 AIS-B spinal cord injury (SCI) that occurred approximately 11 years prior to study enrollment.
Recruitment	Participant T5 and T11 were enrolled in the BrainGate2 pilot clinical trial prior to the design and execution of this study, after meeting inclusion criteria based in part on disease characteristics. Inclusion and exclusion criteria are available online (Clinica|Trials.gov).
Ethics oversight	The BrainGate2 Neural Interface System clinical trial was approved under an Investigational Device Exemption (IDE) by the US Food and Drug Administration (Investigational Device Exemption #G090003 limited by Federal law to investigational use). The Institutional Review Boards of Mass General Brigham/Massachusetts General Hospital, Brown University, Providence VA Medical Center, and Stanford University granted permission for this study. All research was performed in accordance with relevant guidelines/ regulations. All research sessions were performed at the participant's place of residence.

Note that full information on the approval of the study protocol must also be provided in the manuscript.

Field-specific reporting

Please select the one below that is the best fit for your research. If you are not sure, read the appropriate sections before making your selection.

☒ Life sciences ☐ Behavioural & social sciences ☐ Ecological, evolutionary & environmental sciences

For a reference copy of the document with all sections, see nature.com/documents/nr-reporting-summary-flat.pdf

Life sciences study design

All studies must disclose on these points even when the disclosure is negative.

Sample size	No sample-size calculation was performed. Data were collected in two participants to characterize the within-subject changes in chronic intracortical neural recordings during cursor control using a brain-computer interface.
Data exclusions	This study is based on brain-computer interface data collected over a series of months. All days are reported in the study and all relevant data is included.
Replication	This study assessed changes in chronic intracortical neural recordings in two participants. Recurring data were collected across multiple days of performance evaluation spanned across months.
Randomization	Randomization into groups is not relevant for this study - only two participants were included in this study.
Blinding	Blinding is not relevant to this study, which assessed the neural recordings during cursor control using a brain-computer interface in two participants.

Reporting for specific materials, systems and methods

We require information from authors about some types of materials, experimental systems and methods used in many studies. Here, indicate whether each material, system or method listed is relevant to your study. If you are not sure if a list item applies to your research, read the appropriate section before selecting a response.

Materials & experimental systems

n/a	Involved in the study
☒	☐ Antibodies
☒	☐ Eukaryotic cell lines
☒	☐ Palaeontology and archaeology
☒	☐ Animals and other organisms
☒	☐ Clinical data
☒	☐ Dual use research of concern
☒	☐ Plants

Methods

n/a	Involved in the study
☒	☐ ChIP-seq
☒	☐ Flow cytometry
☒	☐ MRI-based neuroimaging

Plants

Seed stocks	*Report on the source of all seed stocks or other plant material used. If applicable, state the seed stock centre and catalogue number. If plant specimens were collected from the field, describe the collection location, date and sampling procedures.*
Novel plant genotypes	*Describe the methods by which all novel plant genotypes were produced. This includes those generated by transgenic approaches, gene editing, chemical/radiation-based mutagenesis and hybridization. For transgenic lines, describe the transformation method, the number of independent lines analyzed and the generation upon which experiments were performed. For gene-edited lines, describe the editor used, the endogenous sequence targeted for editing, the targeting guide RNA sequence (if applicable) and how the editor was applied.*
Authentication	*Describe any authentication procedures for each seed stock used or novel genotype generated. Describe any experiments used to assess the effect of a mutation and, where applicable, how potential secondary effects (e.g. second site T-DNA insertions, mosiacism, off-target gene editing) were examined.*
